# Epidemiology in primary care: opportunities and challenges

**DOI:** 10.1186/2049-3258-73-S1-K2

**Published:** 2015-09-17

**Authors:** Viviane Van Casteren

**Affiliations:** 1 WIV-ISP, Brussels, Belgium

## Background

Primary care-based data are an important source of information on the population's health and care delivery. By its long history of observational studies, peer reviewed and national publications, the Belgian network of Sentinel General Practices (SGP) provides knowledge about epidemiological trends and quality of care. The SGP is a voluntary, paper-based, continuous and representative network. Besides the extensive SGP data, the Health Services Research unit also collects data from GP samples or the total GP population. In the latter, quality of care studies, data are extracted from electronic health records (EHR), combined or not with prompt data input from GPs. In the near future, all GP data will be collected this way. This transition presents opportunities and challenges.

## Opportunities

Large datasets with longitudinal data may be established with less workload for the GPs. EHR-data are linked to single patients with all their health problems or multimorbidity. Estimating the population at risk becomes more precise by using the yearly contact group, adjusted or not for non-attenders.

## Challenges

In 2014, 84% of SGP were using EHR and 75% a certified EHR system. Yet, the regional distribution of EHR systems is highly skewed. In our most recent quality of care study, only 9% of the GPs succeeded in uploading EHR data. Examining the quality of extracted EHR-data is necessary and laborious. We found that only 17% of SGP was always using uniform diagnostic headings to record symptoms and diagnoses in EHR. Moreover, for several health problems there is no code or a combination of codes is needed.

## Conclusions

Efforts are needed to obtain high quality EHR data for research as well as for clinical care. The eHealth roadmap for a global use of e-health services in patient care by 2018 may contribute to this goal.

